# The responsibility to quench thirst by providing drinks when a relative is dying spouses’ experience in specialist palliative home care

**DOI:** 10.1186/s12904-023-01306-1

**Published:** 2023-11-20

**Authors:** Maria Friedrichsen, Nana Waldréus, Micha Milovanovic, Anne Söderlund Schaller, Pier Jaarsma, Tiny Jaarsma

**Affiliations:** 1 Palliative Education and Research Centre, Vrinnevi Hospital, Norrköping, Sweden; 2 Department of Health, Medicine and Caring Sciences, Linköping University, Linköping, Sweden; 3 Department of Neurobiology, Care Sciences and Society, Division of Nursing, Karolinska Institutet, Huddinge, Sweden; 4 Theme Inflammation and Aging, Karolinska University Hospital, Huddinge, Sweden; 5 Department of Internal Medicine, Vrinnevi Hospital, Norrköping, Sweden

**Keywords:** Specialist palliative care, Thirst, Spouses, Thematic analysis

## Abstract

**Background:**

Thirst and dry mouth are common symptoms in terminally ill patients. It is known that family members usually request drips for their dying relative. Few studies have focused on thirst in terminally ill patients and their spouses’ experience of this, leading to a knowledge gap in this area.

**Aim:**

The aim of this study was to explore spouses’ experiences of observing and managing thirst in a dying relative admitted to specialist palliative home care.

**Methods:**

A qualitative interview study with an inductive approach was conducted. Eighteen spouses caring for their husband or wife admitted to specialist palliative home care in different hospitals in Sweden were interviewed. The interviews were transcribed verbatim and analysed with a reflexive thematic analysis.

**Results:**

Three main themes emerged regarding spouses’ experiences of patients’ thirst: Knowledge and views of thirst; Control of fluid intake provides vital information; and Taking charge of their drinking is a life and death responsibility.

**Conclusions:**

Spouses experience a responsibility to serve the dying person with fluids so that they will not get thirsty. It is so obvious and commonplace to them. To be able to fulfil this responsibility, they need to keep track of the patient’s fluid intake and know what quenches thirst. There is a need for research in this area to assist carers and patients in identifying which drinks best quench the patient’s thirst. Interventions are also needed to help provide/make available knowledge on suitable thirst-quenching drinks.

**Supplementary Information:**

The online version contains supplementary material available at 10.1186/s12904-023-01306-1.

## Background

The goal of palliative end-of-life care for terminally ill patients is to prevent and relieve suffering as much as possible as well as the wishes of the patients’ and family members’ [1]. Family members are important in palliative care and especially in home care, as they are closest to the patient and provide much of the care, i.e., family members play a vital role in symptom assessment, monitoring, and delivering therapeutic interventions [2, 3]. This will influence them in multiple ways. Physically, being present 24/7 with all the practical care activities they have to manage [4, 5] and witnessing stressful health events [6]. Psychologically, increased stress and worry about the future [7] supress their own personal needs and feelings while carrying the burden of care [8]. Socially, the patient’s life-threatening illness interferes with social life [7], and spirituality, involved feelings of guilt about the illness in their loved one and about wishing for their death, and about their anger at feeling of abandoned by God [9]. Higher levels of unmet support needs were significantly associated with poorer quality of life [7]. The latter result was also confirmed in another study [10] that reported that all subscales of quality of life were impaired in caregivers of advanced cancer patients, with physical health being the most prominent.

When patients are near the end of life, they may stop eating and drinking or diminish their drinking [11]. A review study found that most family members who experienced diminishing drinking by their dying relative were distressed by it [12]. Feelings of helplessness, guilt and acceptance were present. Some family members responded by protecting the dying relatives from pressure to drink to force them to drink [12].

Common symptoms in patients before death might be pain, fatigue, impaired cognitive functioning, and thirst and dry throat [13]. In the final week of a patient’s life, there is a risk of thirst [14], as the patient may be unable to communicate their needs or may have problems swallowing fluids. Thirst is the subjective sensation of a desire to drink water that cannot be ignored [15]. Carroll [16] described a four-compartment model comprising both primary and secondary thirst: true thirst (osmo-regulated); contextual thirst (e.g., induced by mouth breathing); pharmacological thirst (induced from drugs); and impulsive thirst (daily spontaneous drinking). During the 25–48 h before death, patients drink on average 250 ml of fluids, but this varies significantly (25–1650 ml) [17]. A recent review showed that no studies specifically addressed thirst in patients in specialist palliative care settings [18]. Thirst studies in palliative care were last carried out around 30 years ago, and these studies regarded thirst as a cause of suffering for terminally ill patients [19–21]. A recent experimental study compared ice chips, the usual mouth care from nursing staff, with mini mint ice cubes and found that 86.6% of patients preferred the mini mint ice cubes. For the sensation of thirst, the plain ice chip intervention group rating decreased 1.7 points (*P* < 0.006), and ratings for mint ice cubes decreased 3.4 points (*P* < 0.0001) [22].

However, the view among healthcare professionals in palliative care is that diminishing drinking is a natural part of the process of dying [23], that thirst seldom appears [24, 25], that intravenous (IV) infusion is not necessary and that there is a resistance towards infusions in palliative care due to patients’ side effects [25, 26]. In one study [27], a secondary finding was that both healthcare professionals and family members wondered whether the dying person experienced thirst. The healthcare professionals themselves doubted the answers they gave to the family members “that the patient does not experience thirst”. However, a recent study among nursing assistants showed that there were no routines for questions about thirst [28]. Some of the family members considered it cruel not to treat thirst [27]. Instead, in palliative care, dry mouth is more accepted and treated due to the use of pharmacological drugs [25]. However, it may be difficult to distinguish between thirst and dry mouth. The aim of this study was to explore spouses’ experiences of observing and managing thirst in a dying relative admitted to specialist palliative home care.

## Methods

### Design

Due to a knowledge gap in relation to this phenomenon, “thirst”, a qualitative, reflexive thematic design with an inductive analysis in accordance with Braun & Clarke [29, 30] was used. The study was guided by the Standard for Reporting Qualitative Research (SRQR) [31].

### Sampling and setting

Three cities in Sweden, with populations between 14,000 and 150,000, all of which had specialist palliative home care units, participated. In Sweden, specialist palliative care is defined as care provided by multiprofessional teams with specific knowledge and skills in palliative care [32]. Purposeful sampling was used to achieve a participant mix of different geographic locations, genders and ages (Table 1). The inclusion criteria were being a spouse of a dying relative admitted to specialist palliative care and being fluent in Swedish. A research nurse who met patients in another study in the same project asked spouses to participate. A research invitation comprising a participant information sheet, an expression of interest form and a stamped addressed envelope was sent to spouses within the specialist palliative care units.

**Table 1:** Demographic data of the participating spouses (*n*=18)

**Gender**	
Female/Male	13/5
**Age**	
M	70
Min-max	59-83
**Relationship to the person admitted to palliative care**	
Spouse	18
**Employment status**	
*Retired*	13
*Working*	5
Patients diagnosis*	
*Cancer*	
Gastrointestinal	7
Urogenital	4
Head neck	1
Pseudomyxoma peritonei	1
*Other diagnosis*	
Heart failure	3
Chronic Obstructive Pulmonary Disease	2
Kidney failure	1
Pulmonary fibrosis	1
Diabetes	2
**Patients stoma**	
Colon	3
Kidney	2
Gastro	1
**Patients nutritional intake**^**a**^	
Eat as usual	8
Reduced the amount of food	5
Do not eat at all	5
Infusions	2
Probe and probing food	1

^a^More than one alternative can be chosen

### Data collection

Data were collected in 2019–2023 using personally recorded interviews (*n* = 16), telephone interviews (*n* = 1) and Zoom meetings (*n* = 1), the latter during the COVID-19 pandemic. The interviews lasted between 7 and 64 min (Md = 20 min). A semi-structured interview guide was developed by the research team and was based on the limited research available in the field. Therefore, the interview guide was designed to capture the spouses’ experiences of the patients’ thirst and its influence on their daily life (Supplementary file). Participants were asked to talk freely, and follow-up questions were occasionally asked to achieve greater clarity. It was used as a checklist to ensure that all questions had been discussed. Two pilot interviews were conducted to test the understanding of the questions and the flow of the questions. A few modifications were made after the pilot interview, such as changes in vocabulary. Fourteen interviews were conducted by two research nurses with an MSc, as well as experience within palliative care and interviewing, and four interviews were conducted by a medical student with a special interest in palliative care. Face-to-face interviews were carried out in the spouses’ homes. A professional transcriber transcribed all interviews verbatim, except for four interviews, which the medical student transcribed for educational purposes.

### Data analysis

A reflexive thematic analysis in accordance with Braun & Clarke [29, 30] was used. The design identified, analysed and interpreted patterns of meaning from the qualitative data and used the results to report concepts and assumptions underpinning the data, which are presented in themes.

A six-step process guided the analysis [33]. The analysis started with a familiarisation with the data, generating initial codes of relevance to the research question. Distinctive themes or subthemes were generated, and a thematic map of the initial candidate themes was built to illustrate the relationships. Each potential theme was scrutinised. Then, the themes were defined and named, and each theme and subtheme was expressed in relation to both the dataset and the research question. Multiple extracts were used from the data items that inform a theme to convey the diversity of expressions of meaning across these data items. Data were analysed both manually, on paper (theme mapping), and a Microsoft Word® (California, U.S.A.) (interview transcripts, coding). No new data emerged after 18 interviews, as the same codes and themes reappeared, but no new ones. One author (MF), who is an associate professor with a PhD in palliative medicine and extensive experience in palliative care research and thematic analysis, read all 18 transcripts and conducted the coding and development of themes and subthemes. A preliminary analysis was sent to all co-authors with a number of quotations to give them an opportunity to be critical of the analysis by comparing the inner logic of the analysis, comparing the relationship between themes, subthemes, and analysing text and quotations. Comments were obtained on whether specific quotes fit into a given theme or subtheme as well as suggestions for restructuring the text. For an illustration from coding to final themes, see Table 2.

**Table 2:** Examples of the analysing process

Interview excerpts (interview number, side number and line number)	Generating initial codes of relevance to the research question	Generating subthemes	Defining and naming themes	Questioning and reflection process from co-authors	Themes of meaning Subtheme and main theme
We calculated that there are thirty-four different tablets to be taken every day. In addition, these should be swallowed with water so I do not think she experiences thirst; it is not something we have talked about anyway. 6:3:50-54.	Due to a large amount of medication and associated drinking to swallow them, the spouse do not think that his wife is thirsty.	Beliefs and knowledge of thirst.	Knowledge of thirst	This seems not to cover the whole theme, since it is not only about assessing thirst but also about reasoning on if they could have thirst (e.g. with the drinking for medication). They made an assumption, but did not assess it. This is not assessment but an assumption.	Subtheme: Assumptions about thirst. Main theme: Knowledge and views of thirst.
Although he says he drinks often but, but I do not know, it is me who goes there with the drink, like this. He gets angry when I say that he not drunk more than that ...”Oh, you keep track of that too”, he says. 2:8:158-160.	The spouse tells about a conflict between her and her husband. The husband feels controlled by her constant monitoring of his fluid intake.	Conflicts about not drinking enough and her checking.	Controlling and exhorting leads to conflicts.	To 'nag' has a negative connotation. Perhaps 'to urge', or 'to exhort' are better alternatives?	Subtheme: Controlling and exhorting leads to conflicts Main theme: Control of fluid intake provides vital information
It is clear that if he does not feel thirsty, then I think he is about to shut down. That is how it is. 3:9:176-177.	If he is not thirsty, he will soon die.	If you do not drink, you die of thirst.	Responsibility for drinking		Sub theme: If you do not drink, you die of thirst Main theme: Being responsible for their drinking is a responsibility for life and death
The difference is well when you are lying down and cannot take that drinkable if you are thirsty then. That day, I will probably be close by. 15:20:451-454.	The day when her husband will become bedridden and in need of help, she will take responsibility for his drinking to quench thirst.	Quenching thirst in the future.	Fluids intake in the future.		Subtheme: Fluids intake in the future Main theme: Being responsible for their drinking is a responsibility for life and death

## Results

### Knowledge and views of thirst

Most spouses had not given that much attention to thirst, as they considered it something basic and natural for all humans. For spouses, drinking was closely associated with thirst. Nevertheless, when asking them more about their everyday life, they had some knowledge about thirst expressed in the subthemes: Assumptions about thirst; Patient’s preferences and aversions and Lack of external knowledge sharing creates distress.

#### Assumptions about thirst

Most spouses said that they had not given much thought to whether the ill person felt thirsty or not. However, they knew how to assess thirst, and they used different methods to assess it. In cases where the ill person could verbally communicate their needs, this meant that the spouse could know with certainty whether the person was thirsty. In other cases, respondents relied on their own or shared drinking habits when assessing the person’s thirst or comparing the person’s previous drinking habits. Thirst could also be assessed based on the patient’s behaviour and body language.

*One way is to take the cup and hold it up to her mouth. //If she knocks it away or covers her mouth, she does not want water. Otherwise, I have not thought of anything. However, it must be pretty clear, when she is awake and sees the cup, and I hold it out and she pushes it away, then I know.* I:14.

*Back then, she could not communicate, as she was unconscious. A few days before she could point, and I brought her a drink, and she drank using the straw, like that, and then she swallowed it… and it was fine after a couple of sips. I can imagine that she was thirsty actually. However, she could not express it.* I:17.

Some spouses were unsure whether the patient was thirsty or had a dry mouth. They assumed that they might have a dry mouth because they were taking medication that caused dry mouth as a side effect or had fungus in their mouth. However, most of them could not distinguish between these conditions with certainty, and they mixed them up.

*If you do not drink, as you should, then you get a little dizzy. Therefore, in a way, when I think about thirst, I think a lot about dry mouth.* I:3.

*However, when she is thirsty, mostly it is because she is dry in the mouth.* I:12.

Other spouses described that the patient had so many medicines to take every day, and therefore needed a large amount of fluids, resulting in that the spouses felt that thirst never occurred.

#### Patients’ preferences and aversions

The spouses explained that they had a good understanding of what the patient wanted to drink as well as what they did not like. Preferences varied greatly, but most mentioned that carbonated water or room-temperature or ice-cold water was the best drink to quench thirst. Milk, ice cream, tea and sweet soft drinks were also mentioned. Spouses also knew what the patient was unable to drink, for example, that the patient had an aversion to coffee, water, nutritional drinks or acidic drinks such as juice.

*He takes a sip of water, or now that he has a craving, he wants Fanta all of a sudden. Yes, and then Coca-Cola or something like that, so he has to take a swallow so that it is something other than water. Otherwise, he wants cold water, so we have bottles in the refrigerator, small bottles of cold water and then we change. Therefore, he always has access to something, water.* I:15.

*The carbonated water gives her pain in her mouth. Although she tries anyway…like…the bubbles…uh, are unpleasant… it hurts a bit. Not so much that she screams aloud. No, it is just not nice.* I:12.

*She does not like nutritional drinks because they are far too sweet, so disgustingly sweet that she will not take them. I:6*.

#### Lack of external knowledge sharing creates distress

The spouses had not received any information or knowledge from the health or community care services about thirst or how to quench it. The reason they gave for not seeking help themselves was that the question of thirst had not been on the agenda before. In addition, drinking was something basic that every human knew how to satisfy. As a substitute, they experimented with different liquids to determine which were easy to drink and good tasting for the ill person. However, they lacked knowledge on how to quench thirst and found it difficult to find something that truly satisfied the patient’s thirst, which was sometimes distressing.

*I: Has anyone informed you about what you can do to quench thirst?*

*R: No, that has not been done. In addition, I do not know if, if it is in any way part of the illness, or the treatment, that it is, it is common to feel thirsty. I have not read or learned anything about that either, any information.* I:11.

*We do not truly have that much (knowledge), I think that the sweet stuff is not really that good, but then again, salt is not that good for the body either, but there is a middle way that we can find that is good. The cranberry juice that he drinks in the morning, it is not that sweet and… well it is not so easy to find something that is…* I: 4.

### Control of fluid intake provides vital information

When the spouses described their daily life, it was clear that some had a need to observe and record the patient’s fluid intake. Control and nagging about drinking more often leads to conflicts between the spouse and the patient. Two subthemes emerged: Observing and recording fluid intake and Controlling and exhorting leads to conflicts.

#### Observing and recording fluid intake

The spouses wanted to have control over the patients’ drinking habits. Some spouses had good control by keeping track of drinking. This was seen as preventive work, as drinking was a sign of vitality. Not drinking at all or very little broke the social norm of ”you ought to drink” and was sometimes described as odd.

*YY drinks about… or uses two decilitres of milk in the morning for the porridge and then… the juice two decilitres now in the morning and… a glass of water two decilitres at lunch and then… afternoon coffee… a glass of juice two decilitres and then… two decilitres in the evening and then he is up to one litre.* I:4.

*He is so strange because he drinks almost nothing. Whenever we go out, like this, I usually bring some water or something like that. He has been drinking very little all his life.* I:13.

Most spouses felt the need to control the volume of drinks the patient was consuming. To do this, some used daily fluid lists where all liquid intake was recorded, while others only ensured that the patient drank, regardless of the volume taken.

*Yes, he gets what he wants to drink. That is how it is. At the same time, he should not drink more than, not more than one and a half litres a day so I keep statistics on how much he drinks so that we do not go over that and it, it’s fine.* I:5.

*Yes, but she has probably drunk approximately 6–7 dl in a day. It is not that much, but it is…well, it is a rough estimate. It could be a litre and it could be half a litre.* I:14.

When spouses talked about their control of the patient’s thirst, they did so by describing the situation the patient had been in earlier in life and their disease journey and the current situation and how they observed that thirst needs could change depending on the state of health. Sometimes this observation provided the spouses with vital information; they processed the new information with the old information to determine the current state of health and what prognosis they could expect.

*So truly, a noticeable change came about six months ago, when he was bad…at the end of the summer, beginning of the autumn. During… after a few months, he said to himself ”I do not understand why I am so thirsty”. He drank a lot of chocolate milk, just poured it down his throat…* I:3.

*However, she used to drink. Except for the last, well, the last three days, it has been bad. In addition, for a while before, when she was in the hospital, a few, a couple of times, she did not drink much, but then, since then, she’s been drinking quite a lot.* I:14.

#### Controlling and exhorting leads to conflicts

The spouses reminded the patient to eat or drink more or less, which they described sometimes led to conflicts between them, as the patient may feel controlled by them. The spouse described that they wanted to do well, to help the relative feel better, but they still urged them to drink a sufficient amount.

*Although he says he drinks often, I do not know, it is me who goes there with the drink, like this. However, I do not keep a count like that. He gets angry when I say that he has not drunk more than that…”Oh, you keep track of it too”, he says.//We have talked, but we usually end up fighting because he takes it as criticism because I nag him about not drinking enough.* I:2.

*… and I might reprimand him, think about that… because it is true that the heart has to work harder if he drinks more so that… well… but it usually works because then we go to that drinking list and read how much has been consumed and see what we have left.* I:4.

Others had weighed the pros and cons and concluded that the controlling and exhorting had to end from their side, in favour of the ill one’s health and wellbeing and their relationship together.

*Now, when I know that we have a limited amount of time, I have decided… because I can otherwise have a tendency to rather nag a little, and then I finally decided … because now, it does not really matter if my nagging gives us another month or two, because if I keep on nagging the relationship will not be good. So then you rather have to make your choices, then I simply do not say anything. I:17*.

### Being responsible for their drinking is a responsibility for life and death

When the patients’ health deteriorated, most of the spouses considered it their responsibility to provide fluids in order to help, care, and ensure quality of life. Some spouses did not want to think about thirst in the future, as this was not a problem ”yet”. Spouses where the patient was closer to death saw it as necessary to provide water or any other fluid to prolong life. Three subthemes were found: Helping and caring; Fluid intake in the future; and If you do not drink, you die of thirst.

#### Helping and caring

It was clear to spouses that they had to ensure that fluids were available, as they saw it as their responsibility. This was done out of concern for the patient so that they would not have to make the effort to go and get something to drink or feel thirsty. Despite a daily routine, they did not feel that they were sacrificing anything but were happy to help the ill person, as their care for them was greater than their own need for free time. Most of them automatically provided the ill person with fluids at home or, if they had to go somewhere, they made sure they had a bottle of drink with them.

*Yes, he sits most of the time in his armchair in the room and watches TV and then… he calls sometimes ”can I have some water or can I have some juice”…* I:5.

*Then, we have water on the dresser, by the bed (laughter). Moreover, we both get up at night and then you take a sip of water after that, then you go back to sleep again.* I:8.

It was not only the desire to prolong life that made spouses take responsibility for drinking. They also wanted to help and provide the patient with quality of life by giving them what they wanted to drink because these were the last times they would have together.

*Yes, every day now we drink wine with the food. //before, I start cooking, then NN comes and says ”yes, today it is wine”. Yes, most of the time it is wine. In any case, he takes a glass of wine and then makes two sandwiches with caviar. He does that on his own. It is the most delicious thing in the whole day, I understand. For him! Then, he should have it!* I:13.

#### Fluid intake in the future

The spouses were aware of the need for fluid intake for the patient, and some were worried about this in the future, as they had no or little knowledge of how the fluid would be provided if the patient could no longer drink on their own. Would thirsting to death be a possibility? Others felt that thirst was moderate and not something that spouses needed to think about at all. Some were aware of how fluid intake would end and said they were not worried ”yet”. Most spouses found it difficult to think about the future and chose to take one day at a time. This depended on the situation the patient was in and whether he/she had stopped eating food and taking nutritional drinks.

*Then, you have to use the available methods. Yes, those methods, it is intravenous or just sucking on a swab or something. No, I have not thought about that, no, I hope that is a long way off.* I:6.

*I have only heard about people who are dying, who are about to die, that you…you, uh, moisten their lips. Because they are thirsty. That is the only thing I have heard about…about thirst//Also I, uh, I would rather not… I think that now we have a good, a good life. Why should I go and think about all the terrible things that are going to happen in advance?* I:13.

#### If you do not drink, you die of thirst

When the patient was closer to death, spouses were more concerned about their fluid intake. Another reason for taking responsibility for providing the ill person with fluids was to ensure that they lived as long as possible, as fluids were associated with life and thirst to death. Some clearly expressed that food is something that humans can live without for a long time, but without fluids, humans soon die of thirst. Therefore, they felt a responsibility to help ensure that the ill person drank enough or, in the last days of life, moistened their mouth.

*Well, the fluid supply is what is keeping her alive now. Because there is not much more to take out of the body, to get out of the body, it is just skin and bones.//Because we know that water is important. Because she has not eaten anything in a month. Nothing. Yes, but, food… you can live without it for 40 days. However, you cannot live without water for three or four days, yes.* I:14.

*Maybe it would be different if he was bedridden and I am at home taking care of him and he gets dry mouth and so on. Then, I will take more responsibility, go, and get some swabs, you know.* I:17.

## Discussion

The main findings of this study indicate that spouses of dying patients have detailed knowledge of the patient’s drinking habits, have control over ensuring that the patient does not drink too much or too little, and feel a strong sense of responsibility for quenching the patient’s thirst. This may seem strange, as they at the beginning of every interview did not think they had much to say about the patient’s thirst. Patients’ thirst is an integrated but seldom reflected part of spouses’ everyday life. In addition, the interviews shed light on the complex role of spouses in fluid intake management to quench thirst.

Finding a drink that quenched the patient’s thirst was difficult for spouses. They tried different options, and some wanted information from the health system on how to quench their thirst and struggled to find something the patient would accept to drink. As previously mentioned, thirst is not well studied in palliative care [18] so the question remains whether the spouse can receive any advice from the healthcare system. However, dry mouth and how to alleviate it are more studied [18]. Nevertheless, it is challenging for spouses (and others) to understand the difference between dry mouth and thirst.

Spouses observe and sometimes track patients’ drinks because they know that this gives them important information about the patient’s condition. Since they are also the ones who are with the patient 24/7, they are also the ones who have the best control over it. They also feel that it is their responsibility and that to be able to be responsible, they need knowledge about the ill relative’s thirst, which they can only obtain from their home environment by observing and controlling the patient’s drinking. In the very last days of the patient’s life, some even perceive that they are responsible for how long the patient survives. This is a substantial responsibility, but for the spouses, it appears to be a natural task. This can be compared with the philosophy in Levinas’ ethical responsibility [34]. Levinas states that when humans face suffering, it reveals our own humanity within a space of shared vulnerability with “The Other” [35]. Every body part from another human is a metaphor for human vulnerability, that demands our response [36]. Levinas explains that this responsibility cannot be ignored. This can be compared to the results of the current study, as spouses felt responsible for the survival of their loved ones, as they had to provide them with fluids to drink. When the spouse sees the ill person’s face, body or vulnerability, they reflexively react by taking responsibility for the ill person. The responsibility caused by “The Other” asks for altruism even at the cost of the spouse’s everyday life, it involves making sacrifices on “The Other’s” behalf. This can clearly be seen in this study, as the spouses never questioned their responsibility.

This ethical responsibility may also explain why family members often request an IV infusion in palliative care, as they leave parts of their responsibility to the healthcare professionals, believing that the healthcare system should take responsibility for keeping the patient alive or at least relieving symptoms. A study from Japan confirmed that (*n* = 499), when a patient could not eat enough, spouses rated parenteral hydration as the highest need (87.7%) [37]. When family members were asked what they were most afraid of if the patient did not receive an intravenous drip, 67% said they were afraid of the patient feeling thirsty [38]. Another study [39] showed that hydration meant hope and comfort. However, this request for IV infusion was not usual among spouses in the current study. This might be a question of different care cultures as well as traditions and cultures. As previously mentioned, IV infusion brings resistance towards IV infusions in palliative care due to patients’ side effects [25, 26, 40].

In the current study, a spouse’s control of fluids was the way they could keep track of the patient’s health status, albeit in a nonprofessional way, which sometimes led to conflicts between the partners. This was confirmed in Holden’s [41] qualitative study of 14 patients with cancer and their spouses, where they identified that loss of appetite is indeed a source of anxiety and conflict within the family and that the amount of food and fluid taken is used as a barometer of the patient’s overall condition.

Therefore, the question is whether it is possible at all to free spouses from some ethical responsibility or how to help the spouse feel less ethical responsibility in such a difficult situation. These results can support team members in being more active in communication with family members about thirst at the end of life. One alternative is to communicate the prognosis of the ill person so that they understand that forcing someone to eat or drink will not help. At the same time, according to Levinas [34], this ethical responsibility is an unconscious act, where one reacts without thinking. Nevertheless, ethical actions should be able to be influenced by information, which can eventually lead to knowledge that later results in acceptance of the fact that the ill person can no longer drink and that the dying process may start soon. Previously, communication, different understandings regarding the disease process, and the provision of nutrition/hydration have been described to cause conflict between family members and proffesionals [42]. Team members should be aware of feelings of guilt in spouses and take appropriate measures to counteract these feelings. It is important to help spouses explore their feelings regarding the losses they experience. They need to be reassured that the healthcare team is excellent in symptom management for the patient until the end of life. It may also be of help in encouraging spouses who wish to participate in the physical care of their loved one, for example, with oral care. However, whether oral care is the best solution to quench thirst at the last days of life has still not been fully researched.

### Strengths and limitations

One of the weaknesses of this study is that the spouses had patients at different stages at the end of life and that we have no data on when the patient actually died. Some may live for a few months more, while others have only a few days left. In terms of thirst and the patient’s fluid intake, this probably has an impact. The study should therefore be read with this in mind. Instead, repeated interviews over a period would have provided additional insight into how patients’ thirst influences them. Furthermore, the participants in the study were spouses and not family members. This may affect the results, as children, siblings and partners may have different experiences and thoughts about thirst in the dying person. Patients with different diseases may also experience thirst in different ways, which in turn may affect spouses’ views on how thirst affects their daily lives. This study was conducted in one country, Sweden, and it is not obvious that the results can be transferred to other countries.

## Conclusion

Spouses experience a responsibility to serve the dying person with fluids so that they will never be thirsty. As the patient deteriorates, this task becomes more difficult, as the patient can no longer communicate. Our assumption is that more research, mainly intervention studies, is needed to help spouses who are closest to the dying person, but also to ensure that the dying person does not suffer from thirst and to alleviate the dying patient’s symptoms.

### Supplementary Information

**Additional file 1.** Supplementary file. Interview guide for spouses

## Data Availability

The datasets generated and/or analysed in this study are not publicly available, as neither the Ethics Committee nor the participants have given permission to share sensitive data, but are available from the corresponding author upon reasonable request.
