# Supplementary material for: The responsibility to quench thirst by providing drinks when a relative is dying spouses’ experience in specialist palliative home care
Source: BMC Palliat Care. 2023 Nov 20;22:184. doi: 10.1186/s12904-023-01306-1 (PMC10658994; doi:10.1186/s12904-023-01306-1)
Supplement: Supplementary file 1 — Additional file 1. Supplementary file. Interview guide for spouses [file 12904_2023_1306_MOESM1_ESM.docx]

Supplementary file. Interview guide for spouses.

| - Would you like to tell me how your spouse’s illness started? - What does a typical day look like for you right now? - Have you ever experienced thirst? Please tell us! - Do you think that your spouse feels thirsty? If so, how do you deal with it? - How does your spouse’s thirst affect his/her daily life? - How does your spouse’s thirst affect your everyday life? At night? - Do you think about your spouse’s thirst needs now? In the future? - Has anyone talked to you about your spouse’s thirst? - Has anyone informed you about what can be done to quench thirst? - Is there anything that you want to tell me about thirst that we have not asked?   Probing questions: “Please, explain; Please, tell me more; and Why/Why not?; and How?” |
| --- |
